# Enoxaparin sodium prevents intestinal microcirculatory dysfunction in endotoxemic rats

**DOI:** 10.1186/cc11303

**Published:** 2012-04-16

**Authors:** Yu-Chang Yeh, Ming-Jiuh Wang, Chih-Peng Lin, Shou-Zen Fan, Jui-Chang Tsai, Wei-Zen Sun, Wen-Je Ko

**Affiliations:** 1 Department of Anesthesiology, National Taiwan University Hospital, No. 7, Chung-Shan S. Road, Taipei, Taiwan; 2 Graduate Institute of Clinical Medicine, College of Medicine, National Taiwan University, No. 7, Chung-Shan S. Road, Taipei, Taiwan; 3 Center for Optoelectronic Biomedicine, College of Medicine, National Taiwan University, No. 1, Jen Ai Road, Sec 1, Taipei, Taiwan; 4 Department of Traumatology, National Taiwan University Hospital, No. 7, Chung-Shan S. Road, Taipei, Taiwan

## Abstract

**Introduction:**

During severe sepsis or septic shock, activation of the inflammatory and coagulatory systems can result in microcirculatory dysfunction as well as microvascular thrombosis, culminating in multiple organ dysfunction and death. Enoxaparin can inhibit factor Xa and attenuate endothelial damage. The primary purpose of this study was to investigate the effect of enoxaparin on intestinal microcirculation in endotoxemic rats.

**Methods:**

Thirty male Wistar rats were divided into the following three groups: sham operated (OP); lipopolysaccharide (LPS); and LPS + Enoxaparin group. The rats received a midline laparotomy to exteriorize a segment of terminal ileum for microcirculation examination by full-field laser perfusion imager and sidestream dark field video microscope on mucosa, muscle, and Peyer's patch. In the LPS and LPS + Enoxaparin groups, 15 mg/kg LPS was administered intravenously to induce endotoxemia, and 400 IU/kg enoxaparin sodium was also administered in the LPS + Enoxaparin group.

**Results:**

At 240 minutes, the mean arterial pressure was higher in the LPS + Enoxaparin group than in the LPS group (93 ± 9 versus 64 ± 16 mm Hg, *P *< 0.001). Microcirculatory blood flow intensity was higher in the LPS + Enoxaparin group than in the LPS group as follows: mucosa (1085 ± 215 versus 617 ± 214 perfusion unit [PU], *P *< 0.001); muscle (760 ± 202 versus 416 ± 223 PU, *P *= 0.001); and Peyer's patch (1,116 ± 245 versus 570 ± 280 PU, *P *< 0.001). Enoxaparin inhibited LPS-induced reduction in perfused small vessel density and increase in heterogeneity of microcirculation.

**Conclusions:**

Enoxaparin can prevent intestinal microcirculatory dysfunction in endotoxemic rats by preventing microvascular thrombosis formation and maintaining normal mean arterial pressure.

## Introduction

Severe sepsis and septic shock are the leading causes of multiple organ dysfunction and death in patients admitted to ICUs. Although the Surviving Sepsis Campaign guidelines led to a decrease in hospital mortality [1], one-year mortality remains high ranging from 21.5% to 71.9% [2]. Increasing evidence supports the existence of an extensive cross-talk between inflammation and coagulation during sepsis [3], and activation of the inflammatory and coagulation systems and down regulation of endothelial-bound anticoagulant mechanisms can cause disseminated microvascular thrombosis [4]. Microvascular thrombosis can prevent oxygen from reaching tissues, decrease the perfused small vessel density, and increase the spatial heterogeneity of the perfused small vessel [5]. These effects lead to tissue ischemia, organ hypoperfusion and further, multiple organ dysfunction and death [6-8].

Early intestinal microcirculatory dysfunction has been observed even in normotensive sepsis [9] and it may lead to complications such as altered intestinal motility [10], mucosa barrier disruption, bacterial translocation [11], and multiple organ dysfunction syndrome [12]. Therefore, the intestinal microcirculation provides an excellent site to investigate sepsis-related microcirculatory dysfunction [13,14]. Many advanced techniques have been developed to investigate microcirculation. A full-field laser perfusion imager can be used to quantitatively measure microcirculatory blood flow intensity [15]. A sidestream dark-field (SDF) video microscope has been used to visualize the small vessel and can calculate the small vessel density, microvascular flow index, and heterogeneity of microcirculation [8].

Enoxaparin sodium is a low-molecular-weight heparin. Its high-affinity fraction of heparin sulfate inhibits factor Xa by catalyzing its binding to antithrombin. It can prevent microvascular thrombosis and attenuate endothelial damage in endotoxemic rats [16]. In the present study, we hypothesized that enoxaparin can prevent microcirculatory dysfunction during severe sepsis and septic shock by reducing microvascular thrombosis. The primary purpose of this study was to investigate the effect of enoxaparin on intestinal microcirculation in endotoxemic rats by application of the full-field laser perfusion imager and the SDF video microscope.

## Materials and methods

A total of 30 male Wistar rats (body weight 250 ± 50 g; Biolasco Taiwan Co., Taipei, Taiwan) were used in this study, which was approved by the Institutional Animal Care and Use Committee (No. 20110308, College of Medicine, National Taiwan University, Taipei, Taiwan). The rats were kept on a 12-hour light/dark cycle and had free access to water and food.

### Anesthesia and surgical procedure

Anesthesia was initiated with 4% isoflurane by using an induction chamber connected to an animal anesthesia machine (Midmark Co., Orchard Park, NY, USA). After the rat was anaesthetized, it was placed supine on an animal warming pad. The anesthesia was maintained using 2% isoflurane by mask. Subcutaneous 0.05 mg/kg atropine sulfate in 10 ml/kg 0.9% NaCl was given to reduce respiratory tract secretion, to block vagal reflexes elicited by manipulation of intestinal viscera, and to replace water vapor loss. Tracheostomy was performed and a 14-G catheter (Surflo; Terumo Corporation, Laguna, Philippines) was inserted into the trachea. Subsequent anesthesia was maintained using 1.2% isoflurane. Polyethylene catheters (PE-50; Intramedic 7411, Clay Adams, Parsippany, NJ, USA) were inserted into the right common carotid artery and external jugular vein. The right common carotid artery catheter was used to continuously monitor arterial blood pressure and heart rate. A continuous infusion of 8 ml/kg/hr 0.9% NaCl was given as maintenance fluid supplement via the external jugular vein catheter. The body temperature was continuously monitored. A three cm long midline laparotomy was performed to exteriorize a segment of terminal ileum (about 6 to 10 cm proximal to the ileocecal valve). A two cm section was performed on the anti-mesenteric aspect of the intestinal lumen using a high frequency desiccator (Aaron 900; Bovie Aaron Medical, St. Petersburg, FL, USA) to carefully expose the opposing mucosa for microcirculation examination [17]. Nearby intestinal muscle and Peyer's patch were also identified for microcirculation examination. The rats were observed for a 15-minute stabilization period.

### Grouping and protocol

The 30 rats were divided into the following three groups: 1, Sham OP; 2, LPS; and 3, LPS + Enoxaparin. After the stabilization period, the time was set to 0 minutes. In the LPS and LPS + Enoxaparin groups, a one-minute bolus injection of 15 mg/kg LPS (Escherichia coli, O127:B8; Sigma-Aldrich Co., St. Louis, MO, USA) in 3 ml/kg 0.9% saline was given intravenously to induced endotoxemia [17], then a one-minute bolus injection of 400 IU/kg enoxaparin sodium in 2 ml/kg 5% dextrose was given in the LPS + Enoxaparin group. In the Sham OP and LPS groups, 2 ml/kg 5% dextrose was administered intravenously. At 240 minutes, blood samples were obtained from the right common carotid artery catheter for laboratory analysis. Euthanasia was performed by exsanguination cardiac arrest under anesthesia.

### Microcirculation examination

A full-field laser perfusion imager (MoorFLPI, Moor Instruments Ltd., Devon, UK) was used to continuously quantitate the microcirculatory blood flow intensity [15,18]. This imager uses laser speckle contrast imaging, which exploits the random speckle pattern that is generated when tissue is illuminated by laser light. The random speckle pattern changes when blood cells move within the region of interest (ROI). When there is a high level of movement (fast flow), the changing pattern becomes more blurred, and the contrast in that region reduces accordingly. Therefore, low contrast is related to high flow and high contrast to low flow. The contrast image is processed to produce a 16-color coded image that correlates with blood flow in the tissue such as blue is defined as low flow and red as high flow. The microcirculatory blood flow intensity of each ROI was recorded as Flux with perfusion unit (PU), which is related to the product of average speed and concentration of moving red blood cells in the tissue sample volume. The images were recorded and analyzed in real time by the MoorFLPI software version 3.0 (Moor Instruments Ltd.). Three separate ROIs were established on mucosa, muscle, and Peyer's patch. The microcirculatory blood flow intensities among the three groups were compared at the following time points: 0, 60, 120, 180, and 240 minutes.

At 240 minutes, the SDF video microscope (MicroScan, Microvision Medical, Amsterdam, The Netherlands) was used to investigate total small vessel (less than 20 *μ*m) density, blood flow classification of each small vessel, perfused small vessel density, microvascular flow index (MFI), and heterogeneity index (HI) [19]. This SDF imaging device illuminates the tissues with polarized green light and measures the reflected light from the tissue surface. Both superficial capillaries and venules can be visualized because the scattered green light is absorbed by the hemoglobin of the red blood cells contained in these vessels. At each time point, three continuous image sequences (10 seconds) were digitally stored for each measured site and data of the three images were averaged for statistics. The images were analyzed using automated analysis software (AVA 3.0, Academic Medical Center, University of Amsterdam, Amsterdam, The Netherlands). Total small vessel density was automatically calculated by the software. A semi-quantitative method was used to classify the blood flow of each small vessel as follows: (0) absent (no flow or filled with microthrombosis), (1) intermittent flow (absence of flow for at least 50% of the time), (2) sluggish flow, and (3) continuous flow [19]. Small vessels with blood flow classified as (2) and (3) were considered as perfused small vessels, and the perfused small vessel density was automatically calculated. To calculate MFI score, the image was divided into four quadrants and the same ordinal scale (0 to 3) was used to assess blood flow in each quadrant. The MFI score represents the averaged values of the four quadrants. The HI was calculated as the highest MFI minus the lowest MFI divided by the mean MFI across the three images of each measured site at a certain time point [19]. The analyses were done by a single investigator who was blinded to grouping.

### Statistical analysis

Data were expressed as mean (standard deviation) and analyzed with statistical software (SPSS 19; IBM SPSS, Chicago, IL, USA). The study was powered (*n *= 10 rats per group) to detect a 20% difference in microcirculatory blood flow intensity in intestinal mucosa among the three groups at 240 minutes, with an alpha level of 0.017 (two-tailed) and a beta level of 0.2 (80% power), assuming a control intensity of 1,200 ± 160 PU. Hemodynamic, body temperature, and microcirculatory blood flow intensity were analyzed with repeated measurement analysis of variance followed by Tukey or Dunnett's T3 multiple comparison tests. Total small vessel density, perfused small vessel density, proportion of perfused small vessels and HI were analyzed with one-way analysis of variance followed by post hoc analysis with Tukey or Dunnett's T3 test. Data of MFI were expressed as median (interquartile range) and analyzed with the Kruskal-Wallis test, followed by post hoc Mann-Whitney analysis with adjustment for multiple comparisons. The error bars in all figures represent the 95% confidence intervals of the mean values. A *P *value < 0.05 was considered to indicate a significant result.

## Results

### Enoxaparin prevented reduction in mean arterial pressure

**Figure 1:**
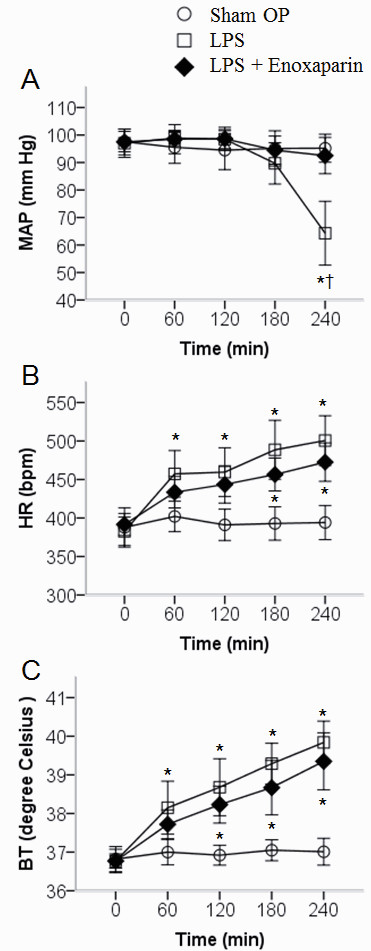
**Hemodynamic data and body temperature**. (**A**) Mean arterial pressure (MAP). (**B**) Heart rate (HR). (**C**) Body temperatures (BT). Circle: Sham OP group; square: LPS group; diamond: LPS + Enoxaparin group, *n *= 10 in each group. *P < 0.05 compared with the Sham OP group; ^†^*P *< 0.05 compared with the LPS + Enoxaparin group. LPS, lipopolysaccharide.

Enoxaparin inhibited LPS-induced reduction in mean arterial pressure (Figure 1A). At 240 minutes, the mean arterial pressure was higher in the LPS + Enoxaparin group than in the LPS group (93 ± 9 versus 64 ± 16 mm Hg, *P *< 0.001). Neither heart rate nor body temperature was significantly different between the LPS group and the LPS + Enoxaparin group (Figure 1B and 1C).

### Enoxaparin inhibited LPS-induced reduction in microcirculatory blood flow intensity

**Figure 2:**
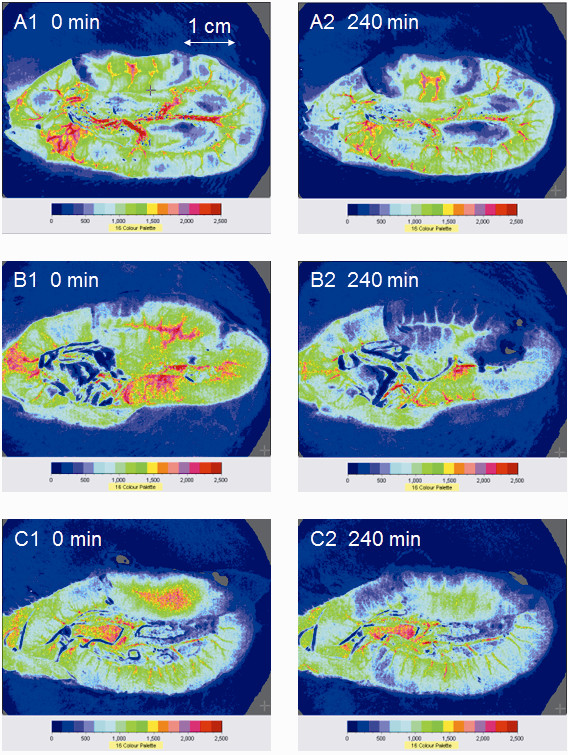
**Images of microcirculatory blood flow intensity of terminal ileum obtained by the full-field laser perfusion imager**. Images for each group are as follows: (**A**) Sham OP group, (**B**) LPS group and (**C**) LPS + Enoxaparin group. LPS, lipopolysaccharide.

**Figure 3:**
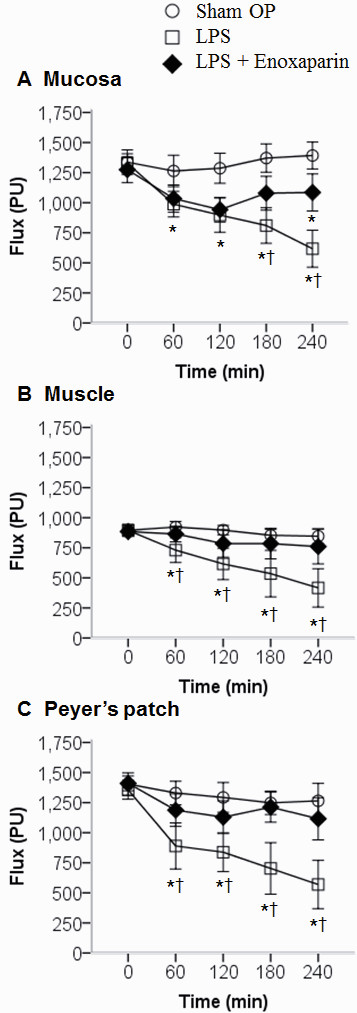
**Comparison of microcirculatory blood flow intensity of the terminal ileum**. Circle: Sham OP group; square: LPS group; diamond: LPS + Enoxaparin group, *n *= 10 in each group. *P < 0.05 compared with the Sham OP group; ^†^*P *< 0.05 compared with the LPS + Enoxaparin group. LPS, lipopolysaccharide.

Examples of the images of microcirculatory blood flow intensity, as obtained by the full-field laser perfusion imager, are shown in Figure 2. Enoxaparin inhibited the LPS-induced reduction in microcirculatory blood flow intensity (Figure 3). At 240 minutes, the microcirculatory blood flow intensity was higher in the LPS + Enoxaparin group than in the LPS group as follows: mucosa (1,085 ± 215 versus 617 ± 214 PU, *P *< 0.001); muscle (760 ± 202 versus 416 ± 223 PU, *P *= 0.001); and Peyer's patch (1,116 ± 245 versus 570 ± 280 PU, *P *< 0.001).

### Enoxaparin inhibited LPS-induced reduction in perfused small vessel density and increase in heterogeneity in microcirculation

**Figure 4:**
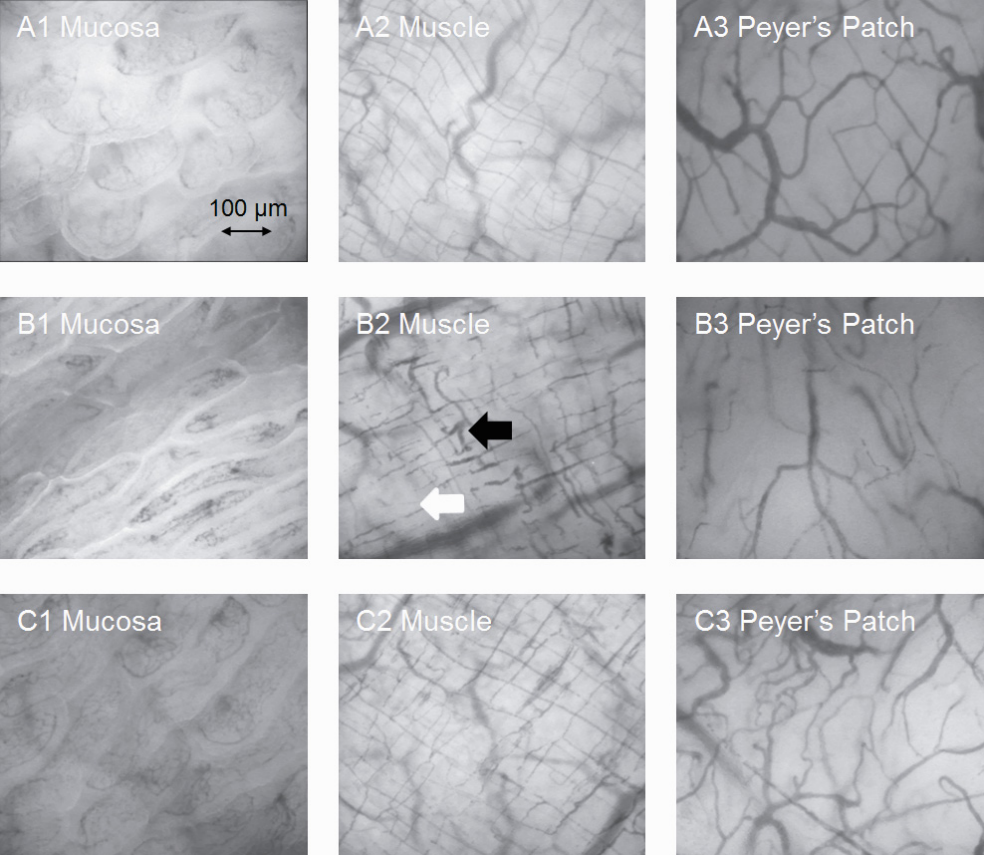
**Images of microcirculation in terminal ileum obtained by the sidestream dark field (SDF) video microscope at 240 minutes**. Images for each group are as follows: (**A**) Sham OP group, (**B**) LPS group and (**C**) LPS + Enoxaparin group. There are small vessels microthrombosis (black arrow), and some small vessels are absent (white arrow) in the LPS group. LPS, lipopolysaccharide.

**Figure 5:**
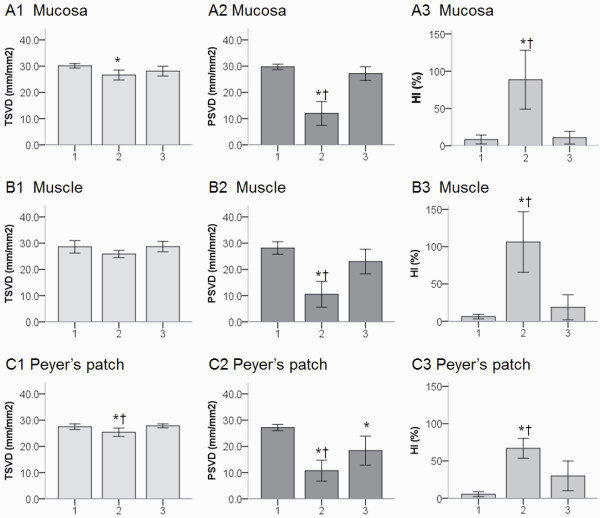
**Comparison of total and perfused small vessel density and heterogeneity index of terminal ileum at 240 minutes**. 1: Sham OP group, 2: LPS group and 3: LPS + Enoxaparin group. *P < 0.05 compared with the Sham OP group; ^†^*P *< 0.05 compared with the LPS + Enoxaparin group. HI, heterogeneity index; LPS, lipopolysaccharide; PSVD, perfused small vessel density; TSVD, total small vessel density.

Examples of the images of intestinal microvasculature, as obtained by the SDF video microscope at 240 minutes, are shown in Figure 4 and Additional file 1, 2, 3, 4, 5 and 6. The total and perfused small vessel density in the LPS group decreased during the experiment (Figure 5), but the difference of total small vessel density in intestinal muscle was not significantly different between the Sham OP group and LPS group. Enoxaparin greatly inhibited the LPS-induced decrease in perfused small vessel density at 240 minutes. The blood flow of many small vessels in the LPS group was absent due to microthrombosis formation. The HIs were higher in the LPS group than in the LPS + Enoxaparin group. The microvascular flow indexes were higher in the LPS + Enoxaparin group than in the LPS group (Table 1).

**Table 1:** Microvascular flow index

Group	Mucosa	Muscle	Peyer's patch
Sham OP	2.9 (2.7-3.0)	2.8 (2.8-3.0)	2.9 (2.8-3.0)
LPS	1.1 (0.5-1.7) *^†^	0.6 (0.5-1.5)*^†^	1.1 (0.9-1.6)*^†^
LPS + Enoxaparin	2.9 (2.7-3.0)	3.0 (2.1-3.0)	2.5 (1.4-3.0)

Data are presented as median (interquartile range). *P < 0.05 compared with the Sham OP group; *^†^P *< 0.05 compared with the LPS + Enoxaparin group. LPS, lipopolysaccharide.

## Discussion

This study shows that enoxaparin can prevent intestinal microcirculatory dysfunction in endotoxemic rats. The evidence is that the microcirculatory blood flow intensity, perfused small vessel density and microvascular flow index were higher in the LPS + Enoxaparin group than in the LPS group. We also found that the blood flow of many small vessels in the LPS group was absent due to microthrombosis formation and that the HIs were higher in the LPS group than in the LPS + Enoxaparin group. These findings indicate that enoxaparin can reduce microvascular thrombosis.

Maintaining adequate and homogeneous perfused small vessel density is very important to avoid tissue hypoperfusion [20]. There are two important pieces of evidence to support the finding that enoxaparin can inhibit the LPS-induced reduction in perfused small vessel density. First, the small vessels should be patent for perfusion. During severe sepsis and septic shock, microvascular thrombosis can obstruct the flow in small vessels and prevent oxygen from reaching the surrounding tissues. Moreover, microvascular thrombosis can direct the microcirculatory blood flow to those small vessels remaining patent and this will lead to microcirculatory shunting [21]. The reduction in microvascular thrombosis and lower HI in the LPS + Enoxaparin group support the conclusion that enoxaparin can maintain adequate and homogeneous perfused small vessels density.

Second, small vessels require adequate arterial pressure for sufficient perfusion. During severe sepsis and septic shock, LPS may activate overt immune and inflammatory responses, which can result in hypovolemia by capillary leakage of fluid and protein [22], cause pathological vasodilation by nitric oxide production [23], and decrease cardiac contractility by myocardial suppression [24]. These derangements can lead to hypotension. The finding that mean arterial pressure remained normal in the LPS + Enoxaparin group indicates that enoxaparin can maintain adequate perfused small vessel density. The mechanism for this protective effect may be related to the anti-inflammatory effect of low molecular weight heparin, which was revealed in previous studies [25-27]. Iba and colleagues [25] demonstrated that not only the improvement of coagulation disorder but also the regulation of circulating levels of pro-inflammatory cytokines may play a role in the mechanism to preserve the organ dysfunction in LPS-challenged rats. Moreover, they also found that enoxaparin protects against endothelial damage by preventing leukocyte adhesion in endotoxemic rats [16]. Many observations support the finding that endothelial activation and dysfunction play a pivotal role in microcirculatory dysfunction during sepsis [28-30]. This may be one of the mechanisms of microcirculatory dysfunction that enoxaparin can prevent.

Compared with a lower LPS concentration rat model, the rats in this study experienced a normotensive endotoxemia (0 to 180 minutes) to shock status (240 minutes). In Figure 3, we can notice that microcirculatory dysfunction deteriorated early at 60 minutes. Consistent with this finding, previous studies have demonstrated that microcirculatory flow alterations can occur in the absence of global hemodynamic derangements [31,32]. The advantage of our rat model is quick investigation of microcirculatory dysfunction within four hours. For exclusive focus on microcirculation for a longer period, a lower LPS concentration rat model is suggested [33]. This rat model has several limitations. First, as in other endotoxemic rat models, early treatment does not reflect the clinical situation [34]. The effect of post-LPS treatment requires further investigation. Second, two rats in the LPS + Enoxaparin group had minor bleeding from surgical wounds in the intestine which were quickly stopped after electrocoagulation using a high frequency desiccator. Although previous study revealed that enoxaparin attenuates endothelial damage with less bleeding compared with unfractionated heparin [16], the bleeding complications should be followed up in other conditions such as late stage of sepsis or prolonged use of enoxaparin. Third, there was still a little small vessel microthrombosis in the LPS + Enoxaparin group. This might be due to an incomplete effect of enoxaparin or thrombin inhibitors induced clotting [35].

## Conclusions

In summary, this study shows that enoxaparin can prevent intestinal microcirculatory dysfunction in endotoxemic rats. Enoxaparin inhibits the LPS-induced reduction in perfused small vessel density and increase in heterogeneity of microcirculation by preventing microvascular thrombosis formation and maintaining normal mean arterial pressure. Besides preventing microvascular thrombosis, enoxaparin may modulate inflammation and reduce endothelial dysfunction. Combining these effects, further studies may be warranted to establish the value and role of enoxaparin in early resuscitation of microcirculatory dysfunction in patients with severe sepsis and septic shock.

## Key messages

• Enoxaparin can prevent intestinal microcirculatory dysfunction in endotoxemic rats.

• Enoxaparin inhibits LPS-induced reduction in perfused small vessel density and increase in heterogeneity of microcirculation by preventing microvascular thrombosis formation and maintaining normal mean arterial pressure.

## Abbreviations

HI: heterogeneity index; LPS: lipopolysaccharide; MAP: mean arterial pressure; MFI: microvascular flow index; PSVD: perfused small vessel density; PU: perfusion unit; SDF: sidestream dark-field; TSVD: total small vessel density.

## Competing interests

The authors declare that they have no competing interests.

## Authors' contributions

YCY participated in the study design, performed animal studies, interpreted the data, and drafted the manuscript. WJK, CPL and JCT planned the experimental design and interpreted the data. SZF and WZS participated in the study design and coordinated the research group. MJW interpreted the results and reviewed the manuscript. All authors read and approved the final manuscript.

## Supplementary Material

Additional file 1 **Microcirculation video of intestinal mucosa of Sham OP group**. Click here for file

Additional file 2 **Microcirculation video of intestinal muscle of Sham OP group**. Click here for file

Additional file 3 **Microcirculation video of intestinal mucosa of LPS group**. Click here for file

Additional file 4 **Microcirculation video of intestinal muscle of LPS group**. Click here for file

Additional file 5 **Microcirculation video of intestinal mucosa of LPS + Enoxaparin group**. Click here for file

Additional file 6 **Microcirculation video of intestinal muscle of LPS + Enoxaparin group**. Click here for file
